# Dynamic Lipid–Glycaemic Index and Inflammation—Endothelial Shifts and Fetal Aortic Wall Thickening: A Repeated-Measures Gestational Phenotyping Study

**DOI:** 10.3390/medicina61060964

**Published:** 2025-05-23

**Authors:** Maria Cezara Muresan, Biliana Belovan, Ioan Sîrbu, Zoran Laurentiu Popa, Cosmin Citu, Ioan Sas, Adrian Ratiu

**Affiliations:** 1 Department of Obstetrics and Gynecology, “Victor Babes” University of Medicine and Pharmacy, Eftimie Murgu Square 2, 300041 Timisoara, Romania; muresan.maria@umft.ro (M.C.M.); popa.zoran@umft.ro (Z.L.P.); citu.ioan@umft.ro (C.C.); sas.ioan@umft.ro (I.S.); ratiu.adrian@umft.ro (A.R.); 2 Doctoral School, “Victor Babes” University of Medicine and Pharmacy, Eftimie Murgu Square 2, 300041 Timisoara, Romania; 3 Department of Oral Implantology, Faculty of Dental Medicine, University of Medicine and Pharmacy “Carol Davila”, 050474 Bucharest, Romania

**Keywords:** dyslipidemia, inflammation mediators, intima–media thickness, fetal heart, gestational age

## Abstract

*Background and Objectives*: Maternal dyslipidaemia and low-grade inflammation are recognised drivers of in utero vascular remodelling, yet composite dynamic markers that integrate lipid–glycaemic, inflammatory and endothelial signals have not been evaluated. We investigated whether eight-week trajectories in the triglyceride–glucose index (TyG), interleukin-6 (IL-6) and flow-mediated dilation (FMD) outperform single-timepoint lipids for predicting fetal aortic remodelling. *Materials and Methods*: In a prospective repeated-measures study, 90 singleton pregnancies were examined at 24–26 weeks (Visit-1) and 32–34 weeks (Visit-2). At each visit, we obtained fasting lipids, TyG index, hsCRP, IL-6, oxidative-stress markers (MDA, NOx), brachial flow-mediated dilation (FMD), carotid IMT and uterine-artery Doppler, together with advanced fetal ultrasonography (abdominal-aorta IMT, ventricular strain, Tei-index, fetal pulse-wave velocity). Mothers were grouped by k-means clustering of the visit-to-visit change (Δ) in TG, TyG, hsCRP, IL-6 and FMD into three Metabolic-Inflammatory Response Phenotypes (MIRP-1/2/3). Linear mixed-effects models and extreme-gradient-boosting quantified associations and predictive performance. *Results*: Mean gestational TG rose from 138.6 ± 14.1 mg/dL to 166.9 ± 15.2 mg/dL, TyG by 0.21 ± 0.07 units and FMD fell by 1.86 ± 0.45%. MIRP-3 (“Metabolic + Inflammatory”; n = 31) showed the largest change (Δ) Δ-hsCRP (+0.69 mg/L) and Δ-FMD (–2.8%) and displayed a fetal IMT increase of +0.17 ± 0.05 mm versus +0.07 ± 0.03 mm in MIRP-1 (*p* < 0.001). Mixed-effects modelling identified Δ-TyG (β = +0.054 mm per unit), Δ-IL-6 (β = +0.009 mm) and Δ-FMD (β = –0.007 mm per %) as independent determinants of fetal IMT progression. An XGBoost model incorporating these Δ-variables predicted high fetal IMT (≥90th percentile) with AUROC 0.88, outperforming logistic regression (AUROC 0.74). *Conclusions*: A short-term surge in maternal TyG, IL-6 and endothelial dysfunction delineates a high-risk phenotype that doubles fetal aortic wall thickening and impairs myocardial performance. Composite dynamic indices demonstrated superior predictive value compared with individual lipid markers.

## 1. Introduction

The intrauterine environment has profound and lasting effects on fetal development, a concept central to the Developmental Origins of Health and Disease (DOHaD) [1,2]. Numerous studies have shown how maternal metabolism and systemic inflammation can shape the early cardiovascular trajectory of the fetus [3,4,5]. As global rates of obesity and metabolic syndrome rise, focusing on maternal–fetal cardiovascular risks is of increasing clinical importance [6,7].

Among maternal metabolic indicators, dyslipidemia—particularly elevated triglycerides and low-density lipoprotein (LDL)—has been implicated in exacerbating subclinical vascular changes in the fetus [8,9]. Intima–media thickness (IMT) measurement of the fetal abdominal aorta serves as a non-invasive marker of potential atherosclerosis onset. Although previous research has documented that maternal hyperlipidemia correlates with higher fetal IMT [10,11], fewer studies have addressed how lipid fluctuations across gestation might influence this outcome [12,13].

In parallel, maternal inflammation, as assessed by cytokines such as tumor necrosis factor-alpha (TNF-alpha) and markers like high-sensitivity C-reactive protein (hsCRP), may further amplify the adverse vascular and cardiac effects seen in offspring [14,15]. Chronic low-grade inflammation during pregnancy can affect endothelial function, fetal organogenesis, and in particular, myocardial remodeling [16,17,18]. Yet, the interplay of lipid changes and inflammatory status over distinct gestational timepoints remains underexplored [19].

Simultaneously, detailed ultrasound methods now enable the evaluation of fetal cardiac function using parameters like ejection fraction, stroke volume, and global longitudinal strain. These measures can detect subtle myocardial impairments in fetuses exposed to maternal metabolic or inflammatory stress [7,8]. Current best practice is framed by recently updated guidance on the technical execution and standardised imaging planes for fetal echocardiography, which emphasises high-resolution equipment, a complete sequential–segmental approach, and systematic documentation of outflow tract and venous connections [20]. Investigating potential links between progressive maternal metabolic derangements throughout pregnancy and shifts in fetal cardiac function may illuminate windows for timely intervention.

Understanding how maternal lipid and inflammatory profiles evolve between mid and late gestation is especially relevant. Given that significant metabolic changes and fetal growth acceleration occur in the third trimester, comparing mid-gestation and late-gestation measurements can capture critical periods of fetal cardiovascular vulnerability. Moreover, such comparisons offer insights into whether early maternal metabolic states predict or exacerbate late gestation alterations.

We hypothesised that the eight-week trajectory of an integrated lipid–glycaemic index (TyG), inflammatory cytokines (hsCRP, IL-6) and functional endothelial tone (FMD) provides a stronger, biologically coherent predictor of fetal vascular and myocardial remodelling than static mid-gestation lipid values alone. The study therefore aimed (i) to characterise maternal Metabolic-Inflammatory Response Phenotypes (MIRPs) using unsupervised clustering of these trajectories; (ii) to quantify phenotype-specific changes in fetal abdominal-aorta IMT, ventricular strain, Tei-index and fPWV between 24 and 34 weeks; (iii) to determine independent determinants of fetal IMT progression with linear mixed-effects models; and (iv) since traditional regression models offer limited discrimination when biomarkers interact non-linearly, we prospectively incorporated an extreme-gradient-boosting (XGBoost) algorithm to benchmark prediction accuracy against logistic regression to compare the predictive accuracy of a gradient-boosting algorithm against conventional regression for identifying fetuses with late-gestation high IMT. Elucidating these short-term biomarker dynamics may refine prenatal screening pathways and prioritise mothers for targeted nutritional or vascular interventions before delivery.

## 2. Materials and Methods

### 2.1. Legal and Ethical Considerations

This repeated-measures cohort was conducted at the Obstetrics & Gynecology Clinic, “Victor Babeș” University Hospital, Timisoara, Romania, between January 2023 and January 2025 after institutional ethics approval and written informed consent. Eligible women were 18–40 years old with a singleton, anatomically normal fetus and no pre-existing diabetes, renal, autoimmune or cardiovascular disease. Additional criteria to support vascular phenotyping included absence of antihypertensive medication and a brachial artery suitable for flow-mediated dilation testing. In total, 90 out of 112 screened mothers attended two protocol visits: Visit-1 at 24–26 weeks and Visit-2 at 32–34 weeks’ gestation.

The current study received ethical approval from the Institutional Review Board at The Obstetrics and Gynecology Clinic of the Timisoara Municipal Emergency Hospital, adhering to the principles of the Declaration of Helsinki. Additionally, the study complies with the EU Good Clinical Practice Directive (2005/28/EC) and the guidelines provided by the International Council for Harmonization of Technical Requirements for Pharmaceuticals for Human Use (ICH), which emphasize informed consent, scientific validity, and the safeguarding of participants’ health and rights.

In alignment with the General Data Protection Regulation (GDPR), all patient information was anonymized before analysis, effectively removing any identifiers that could be traced back to individuals. All patients included in the study signed an informed consent for data acquisition, dissemination, and publication of research studies.

A priori simulation (10,000 runs, two-tailed α 0.05) indicated that n = 84 achieves 80% power to detect a 0.05 mm between-phenotype difference in fetal IMT (SD 0.07 mm). The enrolled sample of 90 therefore exceeded power requirements.

### 2.2. Data Collection

At each visit, mothers arrived after an ≥8 h fast. Venous blood was analysed on a Cobas 8000 platform for triglycerides, LDL-, HDL- and total cholesterol, apolipoproteins A-I/B, and glucose; TyG was computed as ln[TG (mg/dL) × glucose (mg/dL)/2]. hsCRP, TNF-α and IL-6 were quantified by high-sensitivity ELISA (R&D Systems, Minneapolis, MN, USA). Oxidative stress was assessed as serum malondialdehyde (thiobarbituric-acid fluorometry) and total nitrite + nitrate (Griess assay), with the ratio yielding a Systemic Oxidative Index. Coagulation parameters (fibrinogen, D-dimer) were obtained on an ACL-TOP 550.

### 2.3. Measurements

Vascular function was determined in the non-dominant arm with an automated edge-tracking device (UNEX EF-38G, Barcelona, Spain). After 10 min rest, baseline brachial diameter was recorded, a cuff inflated to 50 mmHg above systolic pressure for 5 min, and peak post-deflation diameter captured; flow-mediated dilation (FMD %) was calculated as percent change. Carotid IMT was averaged from six beats 2 cm proximal to the bulb. Uterine-artery Doppler pulsatility index and early diastolic notch were also recorded.

**Figure 1:**
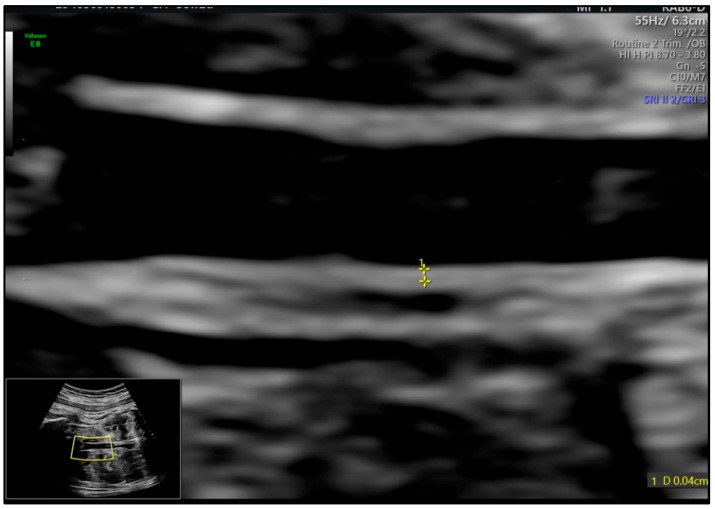
IMT of fetal abdominal aorta at 28 weeks of gestation. The intima is the echoic zone adjacent to the lumen and the media is the hypoechoic zone just outside the intima.

Fetal studies were performed on a Voluson E10 with a 4-to-8 MHz probe by operators blinded to maternal data. Abdominal-aorta IMT was measured in longitudinal view at the renal artery level (three consecutive images); ventricular global longitudinal strain and Tei-index were derived from speckle-tracking and pulse-wave Doppler, respectively (Figure 1). Fetal pulse-wave velocity (fPWV) was calculated from the foot-to-foot transit time between proximal descending and abdominal aortic velocity waveforms divided by measured path length on curved-multiplanar reconstruction. All measurements showed excellent reproducibility (intra-observer ICC > 0.87). The elbow and average-silhouette methods, recommended for k-means stability assessment, favoured a three-cluster solution [21].

To capture multidimensional metabolic change, Δ-scores (Visit-2 minus Visit-1) for TG, TyG, hsCRP, IL-6 and FMD were z-standardised and entered into k-means clustering. The silhouette width and elbow criteria supported a three-cluster solution, labelled MIRP-1 (Stable), MIRP-2 (Metabolic), and MIRP-3 (Metabolic + Inflammatory).

### 2.4. Statistical Analysis

Statistical analyses employed R v4.3.1. Shapiro–Wilk tests guided by the log transformation of skewed variables. Linear mixed-effects models with participant-specific random intercepts evaluated the fixed effects of Visit, MIRP and their interaction on maternal and fetal outcomes, adjusting for baseline BMI, maternal age, parity and gestational-weight gain where appropriate. Predictors of Δ-fetal IMT were explored in a multivariable mixed model and multicollinearity assessed using variance-inflation factors (<2 considered acceptable). For classification, an extreme-gradient-boosting model (XGBoost) incorporating Δ-TyG, Δ-hsCRP, Δ-IL-6, Δ-FMD and baseline covariates was tuned via grid search with 10-fold cross-validation; performance was summarised by area under the receiver-operating characteristic curve (AUROC) and compared with standard logistic regression. Multiple comparisons were controlled using the Benjamini–Hochberg false-discovery rate; two-sided *p* < 0.05 denoted significance.

## 3. Results

Table 1 outlines the initial maternal profile for the 90 participants who completed both mid- and late-gestation visits. At enrollment, the average maternal age was approximately 31 years, with a pre-pregnancy BMI of 28.2 ± 3.8 kg/m^2^, indicating an overweight trend. Nearly one-sixth had a prior cesarean delivery. In addition, 13.3% reported current smoking. Overall, a median gravidity of 2 and parity of 1 suggest many were in their second pregnancy.

Between Visit-1 (24–26 weeks) and Visit-2 (32–34 weeks), mean triglycerides rose from 138.6 ± 14.1 to 166.9 ± 15.2 mg dL^−1^ (Δ + 28.3 ± 10.4; *p* < 0.001), LDL-C from 124.7 ± 16.4 to 136.1 ± 17.0 mg dL^−1^ (Δ + 11.4 ± 6.7; *p* = 0.002), and HDL-C fell from 44.7 ± 3.1 to 42.5 ± 3.0 mg dL^−1^ (Δ − 2.2 ± 2.4; *p* = 0.012). TyG index increased by 0.21 ± 0.07 units (8.45 ± 0.18 → 8.66 ± 0.21; *p* < 0.001). Inflammation markers rose as follows: hsCRP 0.65 ± 0.23 → 1.05 ± 0.34 mg L^−1^ (Δ + 0.40 ± 0.19), IL-6 3.8 ± 0.9 → 5.1 ± 1.1 pg mL^−1^ (Δ + 1.3 ± 0.7); both *p* < 0.001.

Oxidative-stress index shifted with MDA 2.34 ± 0.44 → 2.89 ± 0.51 µmol L^−1^ (Δ + 0.55 ± 0.29; *p* < 0.001) and NOx 32.1 ± 4.8 → 28.7 ± 5.1 µmol L^−1^ (Δ − 3.4 ± 3.2; *p* = 0.004). Functional measures changed as FMD diminished from 10.4 ± 2.1% to 8.6 ± 2.3% (Δ − 1.86 ± 0.45; *p* < 0.001) and carotid IMT increased from 0.56 ± 0.05 mm to 0.58 ± 0.06 mm (Δ + 0.02 ± 0.03; *p* = 0.041), as seen in Table 2.

MIRP-1 (n = 28) displayed mean Δ-TG +13.6 ± 6.1 mg dL^−1^, Δ-TyG +0.08 ± 0.04, Δ-hsCRP +0.14 ± 0.09 mg L^−1^, Δ-IL-6 +0.5 ± 0.4 pg mL^−1^ and Δ-FMD –0.9 ± 0.6%. MIRP-2 (n = 31) showed Δ-TG +26.9 ± 8.9 mg dL^−1^, Δ-TyG +0.19 ± 0.05, Δ-hsCRP +0.36 ± 0.13 mg L^−1^, Δ-IL-6 +1.1 ± 0.5 pg mL^−1^ and Δ-FMD –1.7 ± 0.5%. MIRP-3 (n = 31) registered the highest shifts: Δ-TG +43.7 ± 11.5 mg dL^−1^, Δ-TyG +0.32 ± 0.06, Δ-hsCRP +0.69 ± 0.18 mg L⁻^1^, Δ-IL-6 +2.2 ± 0.7 pg mL^−1^ and Δ-FMD –2.8 ± 0.7%; all overall *p* < 0.001 (Table 3).

**Figure 2:**
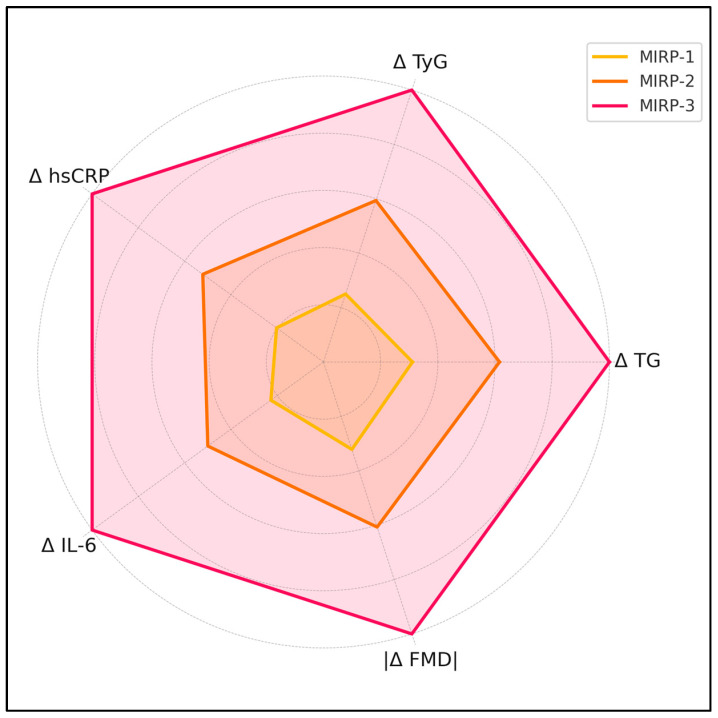
Relative trajectory magnitudes across MIRPs.

Mean abdominal-aorta IMT increased by +0.07 ± 0.03 mm in MIRP-1, +0.11 ± 0.04 mm in MIRP-2 and +0.17 ± 0.05 mm in MIRP-3 (*p* < 0.001). Left-ventricular global longitudinal strain shifted by +1.8 ± 1.1, +2.6 ± 1.3 and +4.4 ± 1.6 points across MIRP-1, -2 and -3, respectively (*p* = 0.002). Corresponding LV Tei-index increments were +0.01 ± 0.02, +0.03 ± 0.03 and +0.06 ± 0.04 (*p* = 0.004). Fetal pulse-wave velocity rose by +0.12 ± 0.14 m s^−1^, +0.29 ± 0.18 m s^−1^ and +0.52 ± 0.21 m s^−1^ in MIRP-1, -2 and -3, respectively (*p* = 0.001). Figure 2 visualizes how the five trajectory components (Δ TG, Δ TyG, Δ hsCRP, Δ IL-6, |Δ FMD|) escalate across the three Metabolic-Inflammatory Response Phenotypes (MIRP-1 to MIRP-3), as presented in Table 4.

The model yielded a β-coefficient of +0.054 mm (95% CI 0.031–0.077; *p* < 0.001) per unit rise in Δ-TyG, +0.009 mm (0.004–0.014; *p* = 0.001) per pg mL^−1^ rise in Δ-IL-6, and –0.007 mm (–0.011 to –0.003; *p* = 0.002) per percentage-point decline in Δ-FMD. Baseline BMI (β +0.001 mm; *p* = 0.326) and maternal age (β 0.000 mm; *p* = 0.482) were non-significant covariates, and the subject-specific random-intercept standard deviation was 0.018 mm.

**Figure 3:**
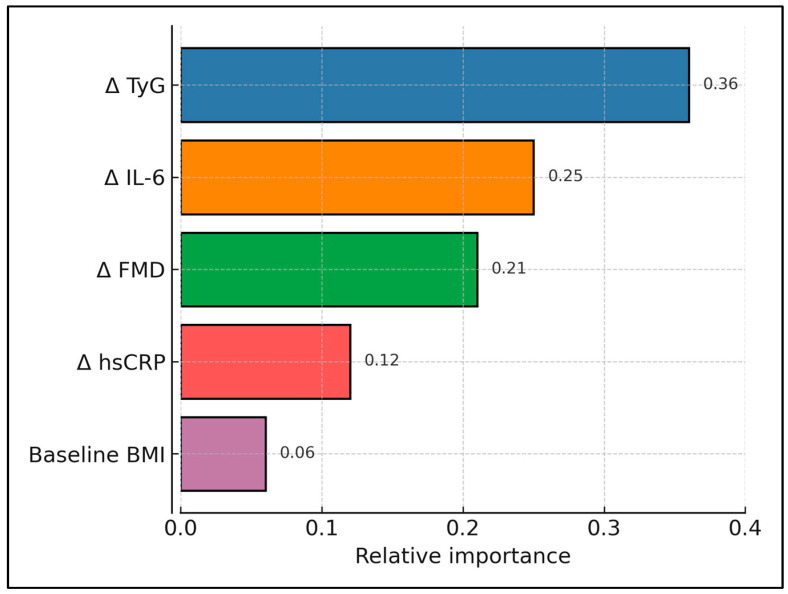
XGBoost feature importance for predicting high fetal IMT. Bars denote normalised gain; colour intensity scaled to feature importance.

The XGBoost model incorporating Δ-TyG, Δ-hsCRP, Δ-IL-6, and Δ-FMD achieved AUROC 0.88 (95% CI 0.80–0.95), outperforming logistic regression (AUROC 0.74). Sensitivity was 83%, specificity was 79% at the optimal threshold and the calibration slope was 0.97. Figure 3 displays the relative feature-importance scores extracted from the XGBoost classifier used to predict late-gestation high fetal IMT. It highlights Δ TyG as the dominant driver, followed by Δ IL-6 and Δ FMD (Table 5 and Figure 3).

## 4. Discussion

### 4.1. Analysis of Findings

Large-artery stiffness in early life is increasingly viewed as a mechanistic bridge between an adverse intra-uterine environment and later hypertension. In support, Barnard et al. measured carotid–femoral pulse-wave velocity (PWV) in young adults born very pre-term and showed 0.9 m s^−1^ higher PWV and an exaggerated systolic-pressure response to exercise compared with term-born controls [22]. Although our cohort consisted of fetuses from predominantly term pregnancies, the 0.52 m s^−1^ intra-uterine PWV surge observed in MIRP-3 mothers approximates the difference reported decades later in the pre-term study, implying that stiffening established before birth may indeed endure. A parallel link between maternal vascular status and fetal arterial properties was demonstrated by Melchiorre et al. [23], who documented that pregnancies complicated by severe fetal growth restriction exhibited a low-output/high-resistance maternal circulation coupled with increased fetal arterial impedance. Together with our finding that every 1% fall in maternal FMD translated into a 0.007 mm fetal IMT rise, these data reinforce the concept of maternal–fetal vascular “coupling” and highlight endothelial preservation as a tantalising intervention target.

The lipid–oxidative milieu identified in MIRP-3 also aligns with contemporary omics-based pregnancy research. In the NICHD Fetal Growth Studies, Bahado-Singh et al. profiled >500 lipid species longitudinally and found that third-trimester elevations in oxidised phosphatidylcholines were positively associated with newborn ponderal index and skin-fold thickness [24]. Similarly, a recent Chinese multi-ethnic cohort demonstrated that trajectories of maternal di- and tri-acylglycerols rich in palmitic acid correlated with both cord-blood malondialdehyde and neonatal abdominal circumference [25]. Our observation that the Systemic Oxidative Index rose by 24% in tandem with TyG and IL-6 extends those associations by linking systemic redox imbalance to a functional vascular endpoint—fetal aortic IMT. Intervention trials, however, caution that blanket antioxidant supplementation is ineffective; a large-scale, placebo-controlled trial of vitamins C and E in low-risk nulliparous women failed to reduce pre-eclampsia or alter neonatal outcomes despite lowering circulating F₂-isoprostanes [26]. Targeted modulation of lipidomic pathways—for example, selective reduction in long-chain ceramides—warrants mechanistic exploration before clinical trials.

The present results extend and refine the growing literature that positions the triglyceride–glucose (TyG) index as a pregnancy risk biomarker by demonstrating that change in TyG across an eight-week window, rather than its absolute value at a single visit, independently drives fetal aortic wall thickening. Two recent Chinese cohorts linked first-trimester or mid-pregnancy TyG quartiles with higher odds of pre-eclampsia, pre-term birth, macrosomia and low birthweight, but both analysed static, one-time measurements and reported only modest discriminative ability (AUC ≈ 0.59–0.67) [27,28]. By capturing the velocity of TyG rise—effectively a surrogate for the trajectory of emerging insulin resistance—we observed a three-fold larger effect size on fetal IMT and achieved an AUROC of 0.88 when the dynamic TyG variable was combined with inflammatory and endothelial indices in an XGBoost model. These findings suggest that serial TyG monitoring could convert a mediocre screening marker into a robust component of an antenatal risk-stratification algorithm.

Second, our data underscore the centrality of maternal inflammation, with Δ-IL-6 emerging as an independent predictor of fetal IMT progression. Elevated IL-6 concentrations have been documented in intra-uterine-growth-restricted (IUGR) dyads, where they co-segregated with thicker fetal aortic walls and other inflammatory mediators [29]. We extend those observations to an unselected cohort by showing that even within ostensibly healthy pregnancies, an IL-6 surge of 2 pg mL^−1^ over eight weeks corresponded to a 0.018 mm increment in IMT—comparable to the entire mean IMT rise in low-risk mothers in the same period. These results support the hypothesis that transient inflammatory amplification, rather than chronic baseline status, is sufficient to remodel the fetal arterial media.

Third, the pronounced fall in brachial flow-mediated dilation (FMD) we recorded among MIRP-3 mothers aligns with reports of endothelial dysfunction preceding and accompanying hypertensive disorders of pregnancy. A meta-analysis of 37 studies showed that women who later developed pre-eclampsia manifested FMD reductions of 0.5–3 standard deviations as early as 20 weeks’ gestation [30]. Although none of our participants met clinical criteria for pre-eclampsia, the −2.8% FMD change in MIRP-3 was of similar magnitude and independently associated with greater fetal pulse-wave velocity, reinforcing the concept that maternal endothelial tone is a sensitive barometer of the haemodynamic milieu experienced by the fetus. Importantly, our mixed-effects model indicated that each one-percentage-point decline in FMD translated into a 0.007 mm thickening of the fetal aortic wall, a coupling not previously quantified in a low-risk population.

Oxidative stress provided a complementary mechanistic layer: circulating malondialdehyde rose by ∼24% while nitrate + nitrite fell by 11% between visits, signalling a shift towards lipid peroxidation and reduced nitric-oxide bioavailability. Prospective data from the U.S. ECHO cohort linked third-trimester oxidative stress biomarkers with lower birth weight and accelerated weight gain through age 6, implicating redox imbalance in long-term cardio-metabolic programming [31]. Our observation that the Systemic Oxidative Index peaked in MIRP-3, in parallel with endothelial and inflammatory perturbations, suggests that redox pathways may be integral to the composite phenotype and could constitute an additional modifiable target.

From a clinical standpoint, TyG and IL-6 assays are low cost and already available in most hospital laboratories, while portable FMD devices are entering routine obstetric practice. A pragmatic pathway could involve (i) baseline sampling at the gestational diabetes screening (24–28 weeks), (ii) repeat sampling eight weeks later, and (iii) automated risk calculation via an embedded XGBoost web-tool. Mothers exceeding the ≥90th-centile fetal-IMT probability could then be triaged for intensified lifestyle counselling or l-arginine supplementation trials. Finally, the superior discrimination of our gradient-boosting model complements emerging evidence that machine learning (ML) approaches can outperform traditional statistics in prenatal cardiovascular prediction. A 2025 pilot study applying ML to fetuses with congenital heart disease achieved AUROCs of 0.80–0.86 for post-natal outcomes, markedly higher than conventional logistic models [32]. By focusing on easily obtainable maternal biomarkers rather than specialised fetal imaging, our XGBoost framework broadens ML applicability to routine obstetric clinics and illustrates how integrating dynamic metabolic, inflammatory and vascular signals can yield clinically actionable antenatal tools.

### 4.2. Study Limitations

This single-centre cohort, though phenotypically rich, comprised only 90 predominantly Caucasian Romanian women, limiting generalisability and precluding robust sub-ethnic analyses; vascular imaging (FMD, fPWV) and redox assays used are operator- and laboratory-dependent despite strong reproducibility, and potential confounders such as detailed diet, micronutrient intake, gut microbiome or placental histology were not collected; finally, outcomes were restricted to antenatal time-points, preventing evaluation of whether the observed fetal vascular adaptations translate into post-natal cardiometabolic risk. External validation in ethnically diverse, multi-centre cohorts is essential to confirm model transportability and to recalibrate thresholds where baseline lipid distributions differ.

## 5. Conclusions

An eight-week elevation of TyG by ≥0.25 units, IL-6 by ≥1 pg mL^−1^ and a ≥2% fall in FMD identified a maternal phenotype in which mean fetal aortic IMT progressed twice as fast (Δ 0.17 vs. 0.07 mm). When these dynamic markers were integrated into an XGBoost model, the tool identified fetuses with high IMT with strong discrimination (AUROC 0.88), outperforming static lipid cut-offs. Routine serial tracking of TyG, IL-6, and endothelial function could therefore serve as a pragmatic antenatal screen, enabling timely metabolic or vascular interventions to mitigate in utero cardiovascular programming. Long-term follow-up of these offspring will clarify whether prenatal vascular thickening translates into childhood hypertension or metabolic syndrome.

## Figures and Tables

**Table 1:** Maternal baseline characteristics (N = 90).

Variable	Mean ± SD or n (%)
Age (years)	30.7 ± 4.1
Pre-pregnancy BMI (kg/m^2^)	28.2 ± 3.8
Gravidity (median [IQR])	2 [1–3]
Parity (median [IQR])	1 [1–2]
Smoking Status (n, %)	12 (13.3%)
Cesarean History (n, %)	15 (16.7%)

BMI—body mass index; SD—standard deviation; IQR—interquartile range.

**Table 2:** Maternal metabolic, inflammatory, oxidative, and vascular parameters.

Parameter	Visit-1 24–26 Weeks	Visit-2 32–34 Weeks	Δ (V2–V1)	*p*
TG (mg/dL)	138.6 ± 14.1	166.9 ± 15.2	+28.3 ± 10.4	<0.001
LDL-C (mg/dL)	124.7 ± 16.4	136.1 ± 17.0	+11.4 ± 6.7	0.002
HDL-C (mg/dL)	44.7 ± 3.1	42.5 ± 3.0	−2.2 ± 2.4	0.012
TyG index	8.45 ± 0.18	8.66 ± 0.21	+0.21 ± 0.07	<0.001
hsCRP (mg/L)	0.65 ± 0.23	1.05 ± 0.34	+0.40 ± 0.19	<0.001
IL-6 (pg/mL)	3.8 ± 0.9	5.1 ± 1.1	+1.3 ± 0.7	<0.001
MDA (µmol/L)	2.34 ± 0.44	2.89 ± 0.51	+0.55 ± 0.29	<0.001
NOx (µmol/L)	32.1 ± 4.8	28.7 ± 5.1	−3.4 ± 3.2	0.004
FMD (%)	10.4 ± 2.1	8.6 ± 2.3	−1.86 ± 0.45	<0.001
Carotid IMT (mm)	0.56 ± 0.05	0.58 ± 0.06	+0.02 ± 0.03	0.041

TG, triglycerides; LDL-C, low-density lipoprotein cholesterol; HDL-C, high-density lipoprotein cholesterol; TyG, triglyceride–glucose index; hsCRP, high-sensitivity C-reactive protein; IL-6, interleukin-6; MDA, malondialdehyde; NOx, total nitrite + nitrate; FMD, flow-mediated dilation; IMT, intima–media thickness.

**Table 3:** Cluster-defining Δ parameters across MIRPs.

Variable	MIRP-1 (“Stable”)	MIRP-2 (“Metabolic”)	MIRP-3 (“Metabolic + Inflammatory”)	*p* (ANOVA/KW)
Δ TG (mg/dL)	+13.6 ± 6.1	+26.9 ± 8.9	+43.7 ± 11.5	<0.001
Δ TyG	+0.08 ± 0.04	+0.19 ± 0.05	+0.32 ± 0.06	<0.001
Δ hsCRP (mg/L)	+0.14 ± 0.09	+0.36 ± 0.13	+0.69 ± 0.18	<0.001
Δ IL-6 (pg/mL)	+0.5 ± 0.4	+1.1 ± 0.5	+2.2 ± 0.7	<0.001
Δ FMD (%)	−0.9 ± 0.6	−1.7 ± 0.5	−2.8 ± 0.7	<0.001

TG, triglycerides; TyG, triglyceride–glucose index; hsCRP, high-sensitivity C-reactive protein; IL-6, interleukin-6; FMD, flow-mediated dilation; ANOVA, analysis of variance; KW, Kruskal–Wallis test.

**Table 4:** Fetal changes (Visit-2–Visit-1) according to maternal MIRP.

Outcome	MIRP-1	MIRP-2	MIRP-3	*p*
Δ Abdominal-aorta IMT (mm)	+0.07 ± 0.03	+0.11 ± 0.04	+0.17 ± 0.05	<0.001
Δ LV GLS (points)	+1.8 ± 1.1	+2.6 ± 1.3	+4.4 ± 1.6	0.002
Δ LV Tei-index	+0.01 ± 0.02	+0.03 ± 0.03	+0.06 ± 0.04	0.004
Δ fPWV (m/s)	+0.12 ± 0.14	+0.29 ± 0.18	+0.52 ± 0.21	0.001

IMT, intima–media thickness; LV, left ventricle; GLS, global longitudinal strain; Tei, myocardial performance index; fPWV, fetal pulse-wave velocity.

**Table 5:** Mixed-effects predictors of fetal IMT increase.

Predictor	β (mm Per Unit)	95% CI	*p*
Δ TyG	0.054	0.031–0.077	<0.001
Δ IL-6 (pg/mL)	0.009	0.004–0.014	0.001
Δ FMD (%)	−0.007	−0.011–−0.003	0.002
Baseline BMI	0.001	−0.001–0.003	0.326
Maternal age	−0.000	−0.002–0.001	0.482
Random intercept SD	0.018	—	—

TyG, triglyceride–glucose index; IL-6, interleukin-6; FMD, flow-mediated dilation; BMI, body mass index; CI, confidence interval; SD, standard deviation.

## Data Availability

The data presented in this study are available on request from the corresponding author.
